# Supplementary material for: The interactome of intact mitochondria by cross-linking mass spectrometry provides evidence for coexisting respiratory supercomplexes
Source: Mol Cell Proteomics. 2017 Dec 8;17(2):216–32. doi: 10.1074/mcp.RA117.000470 (PMC5795388; doi:10.1074/mcp.RA117.000470)
Supplement: Supplemental Data [file supp_RA117.000470_133922_0_supp_23978_fzffwf.zip › spectra_annotation/mito_spectra_annotation/129-1-1-1-3-1.pdf]

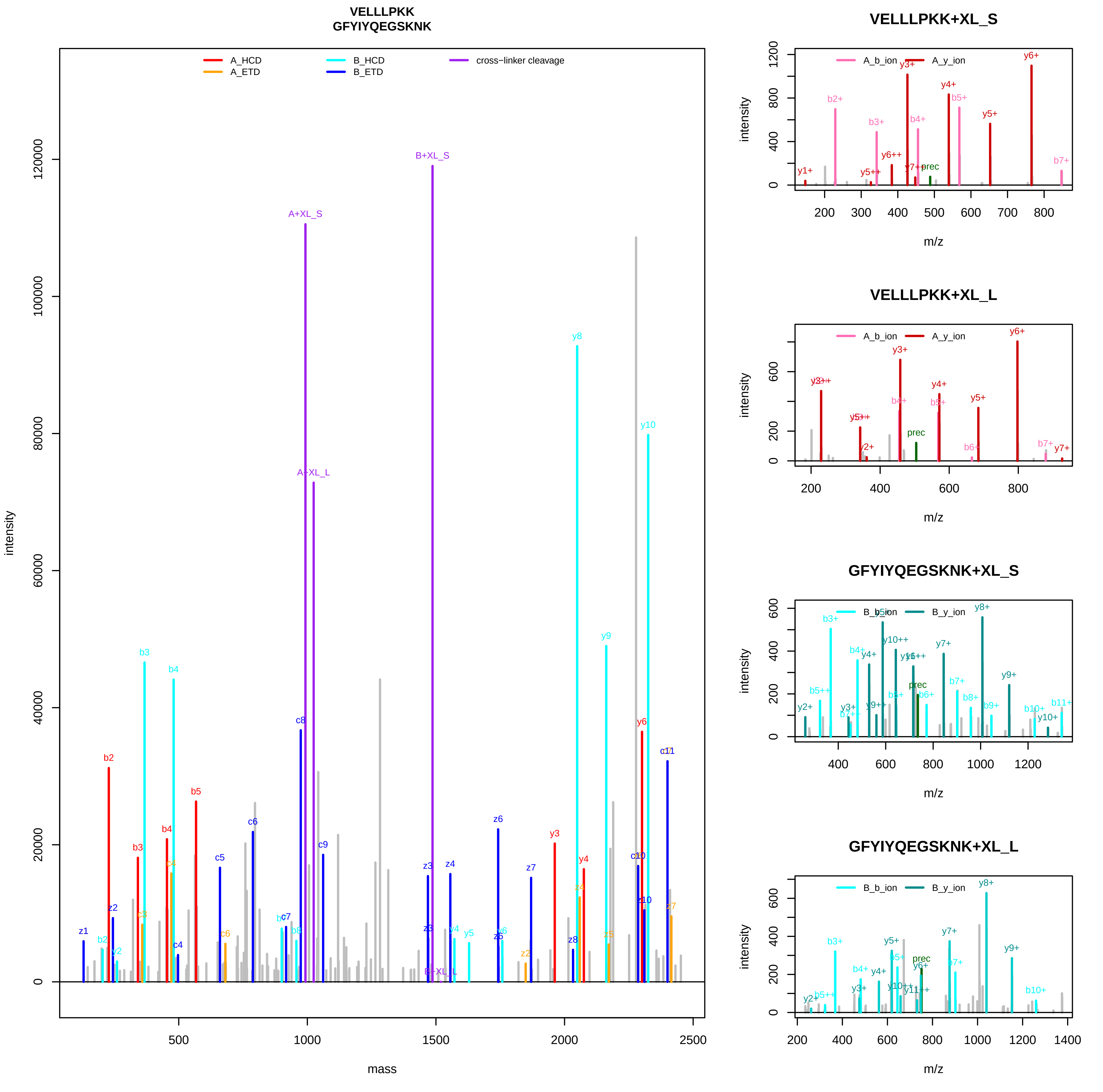
VELLLPKK
GFYIYQEGSKNK
A_HCD
A_ETD
B_HCD
B_ETD
cross−linker cleavage
B+XL_S
A+XL_S
A+XL_L
B+XL_L
y8
y10
y9
y6
c11
c10
z10
z7
b2
b3
b4
b5
c3
c4
c5
c6
c7
c8
c9
z1
z2
y2
z3
z4
y4
y5
z6
y6
z7
z8
z2
y3
y4
z4
z5
intensity
mass
VELLLPKK+XL_S
A_b_ion
A_y_ion
y6+
y3+
y4+
b5+
b2+
b3+
b4+
y5+
y6++
y5++
y7++
prec
y1+
b7+
intensity
m/z
VELLLPKK+XL_L
A_b_ion
A_y_ion
y6+
y3+
y3++
y4+
y5+
b4+
b5+
y5++
y2+
prec
b6+
b7+
y7+
intensity
m/z
GFYIYQEGSKNK+XL_S
B_b_ion
B_y_ion
y8+
b3+
y10++
y7+
b4+
y4+
b5++
prec
b7+
b8+
b9+
y9+
y2+
y3+
b10+
b11+
y10+
intensity
m/z
GFYIYQEGSKNK+XL_L
B_b_ion
B_y_ion
y8+
b3+
y5+
y7+
y9+
b4+
y4+
prec
y6+
b7+
y3+
y10++
y11++
y2+
b5++
b10+
intensity
m/z
